# High glucose induces apoptosis via upregulation of Bim expression in proximal tubule epithelial cells

**DOI:** 10.18632/oncotarget.15491

**Published:** 2017-02-18

**Authors:** Xiao-Qian Zhang, Jian-Jun Dong, Tian Cai, Xue Shen, Xiao-Jun Zhou, Lin Liao

**Affiliations:** ^1^ Department of Endocrinology, Shandong Provincial Qianfoshan Hospital, Shandong University, Jinan, Shandong, China; ^2^ Department of Endocrinology, Qilu Hospital of Shandong University, Shandong, Jinan, China; ^3^ Department of Endocrinology, Shandong Provincial Qianfoshan Hospital, Shandong University of Traditional Chinese Medicine, Jinan, China; ^4^ Department of Medicine, Tai'an Hospital of Traditional Chinese Medicine, Tai'an, Shandong, China

**Keywords:** diabetic nephropathy, high glucose, bim, apoptosis, autophagy, Pathology Section

## Abstract

Diabetic nephropathy is the primary cause of end-stage renal disease. Apoptosis of tubule epithelial cells is a major feature of diabetic nephropathy. The mechanisms of high glucose (HG) induced apoptosis are not fully understood. Here we demonstrated that, HG induced apoptosis via upregulating the expression of proapoptotic Bcl-2 homology domain 3 (BH3)-only protein Bim protein, but not bring a significant change in the baseline level of autophagy in HK2 cells. The increase of Bim expression was caused by the ugregulation of transcription factors, FOXO1 and FOXO3a. Bim expression initiates BAX/BAK-mediated mitochondria-dependent apoptosis. Silence of Bim by siRNA in HK2 cells prevented HG-induced apoptosis and also sensitized HK2 cells to autophagy during HG treatment. The autophagy inhibitor 3-MA increased the injury in Bim knockdown HK2 cells by retriggering apoptosis. The above results suggest a Bim-independent apoptosis pathway in HK2 cells, which normally could be inhibited by autophagy. Overall, our results indicate that HG induces apoptosis *via* up-regulation of Bim expression in proximal tubule epithelial cells.

## INTRODUCTION

Diabetic nephropathy (DN) has been thought as glomerulapathy [1–6]. Recent researches have demonstrated that tubulopathy, especially the apoptosis of proximal tubule epithelial cells, also plays an important role in DN, which occurs earlier than glomerulopathy [7–9]. Intervention of tubulopathy may be better and more effective in DN treatment since it happens in an earlier stage [10]. However, the detailed mechanism of tubulopathy is unclear.

Autophagy is a major catabolic pathway involved in degrading macromolecules and damaged organelles to maintain intracellular homeostasis [11]. It has been revealed that autophagy plays a renoprotective role in animal models of aging or acute kidney injury [12–16]. Researches suggested that targeted therapy to autophagic pathway, attempting to restore autophagy activity may be renoprotective [17–19]. Impairment of autophagy activity leads to apoptosis [20]. Nevertheless, the mechanism of apoptosis is not clear.

The BCL-2 family of proteins regulates apoptosis through a balanced activity of pro- and antiapoptotic family members. Among the BCL-2 family of proteins, Bim is a proapoptotic protein with only one BCL-2 homology (BH3) domain [21]. It plays a significant role in the activation of cell death pathways. Bim expression is increased by high glucose (HG), which promotes apoptosis of retinal pericytes [22]. However, the role of Bim in the development of DN has not been addressed yet.

We designed this study attempting to elucidate possible mechanisms of high glucose induced apoptosis in proximaltubule epithelial cells.

## RESULTS

### High glucose (HG) increases apoptosis in HK2 cells

**Figure 1:**
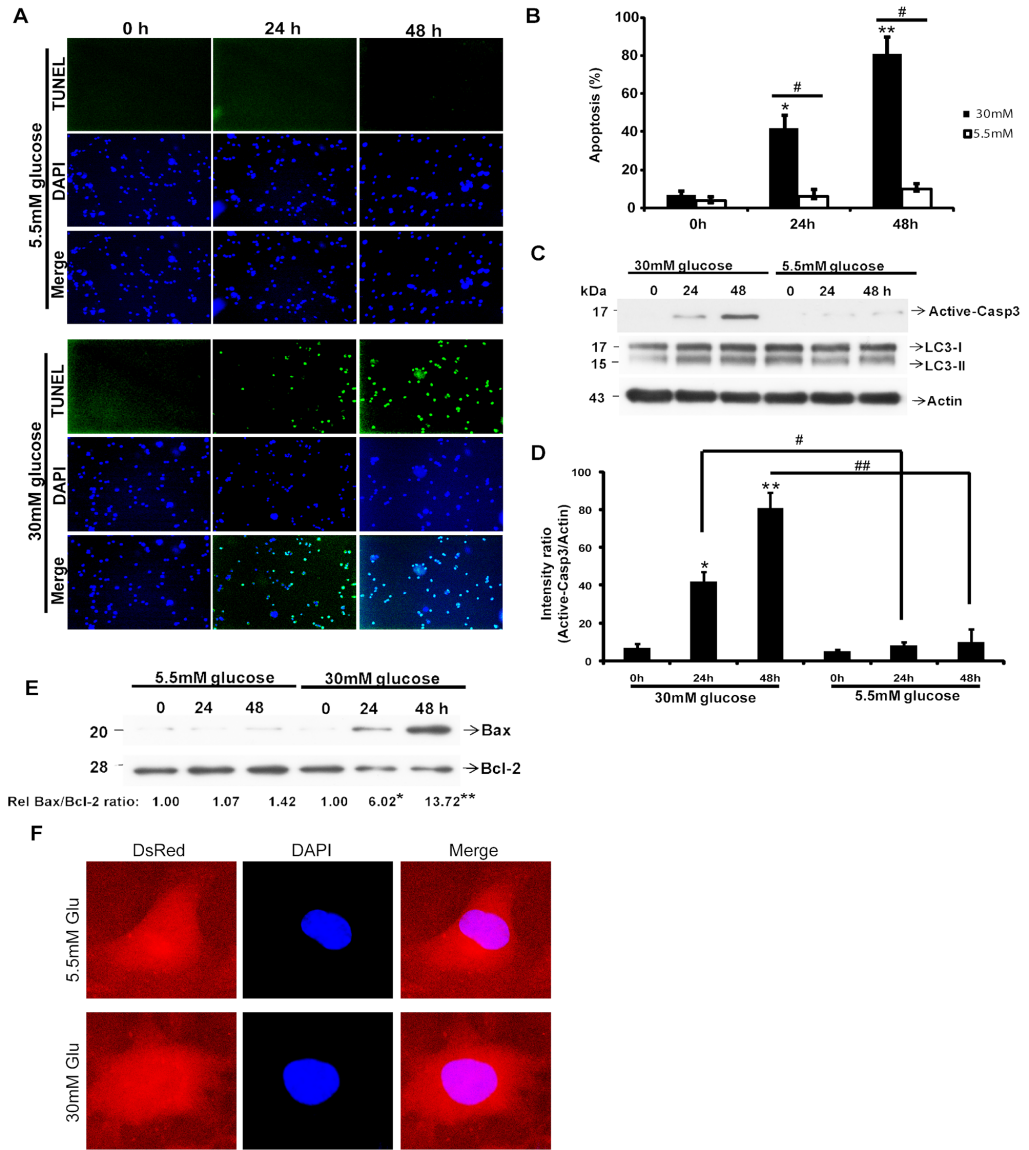
High glucose (HG) induced apoptosis in HK2 cells HK2 cells were treated with 30mM or 5.5mM glucose for indicated times. **A**. Apoptosis was determined by TUNEL staining (green dots) and doubly stained with DAPI (blue dots). **B**. The percent of cell death was quantified by dividing the number of apoptotic nuclei to a population of 1,000 counted cells per condition. Graphs represent means ± s.e.m. *n* = 3; *P < 0.05, **P < 0.01, relative to the 0h group; one-way ANOVA.^#^*p* < 0.05, relative to 5.5mM group; Student's t test. **C**. Imunobloting analysis of Caspase3, LC3 and Actin. **D**. Active-Caspase3/Actin immunoreactivityintensity is quantitated by densitometric analysis, and optical density values are expressed as a ratiobetween the Active-Caspase3 and Actin. Data shown are the mean ± s.e.m. *n* = 3. *P < 0.05, **P < 0.01, relative to the 0h group; one-way ANOVA. ^#^*p* < 0.05, ^##^*p* < 0.01; relative to 5.5mM group; Student's t test. **E**. Protein levels of BAX and BCL-2 were examined by immunoblotting. Quantification of the expressionratio of BAX/BCL-2 is shown with the ratio of 1.0 being assigned to 5.5mM-0h cells. *P < 0.05, **P < 0.01, one-way ANOVA. F, Representative images of HK2 cells transfected with Dsred-LC3-GFP for 48 h. after treated with 5.5 mM or 30mM glucose, cells were fixed followed by microscopy.

To examine the effect of HG on HK2 cells, the cells were treated with glucose at various concentrations at different time courses. The effect of glucose on apoptosis in HK2 cells was evaluated by a TUNEL assay and western blot of active-Caspase3 (active-Casp3). A time-dependent increase ofTUNEL-positive cells in HG-treated (30mM glucose) HK2 cells was observed as compared with the control group (normal glucose, 5.5 mM) (*P* < 0.05, Figure 1A). Quantification for the TUNEL-positive cell is shown in Figure 1B (*P* < 0.05). HG (30mM glucose) treatment also induced a significant time-dependent increase of Active-Caspase 3 (*P* < 0.05, Figure 1C, 1D). BCL-2 protein, an inhibitor of apoptosis, BAX protein, an inducer of apoptosis, both of them determine survival or death after an apoptotic stimulus. Previous studies suggest that the ratio of BAX to BCL-2 is a useful index to evaluate apoptosis. We examined protein levels of BAX and BCL-2 in different treatment groups by western blot, and found that HG markedly increased the ratio of BAX/BCL-2 protein compared with control group (*P* < 0.05, Figure 1E).

Several reports demonstrated that HG may induce autophagy of proximal tubule epithelial cells, we therefore assessed the role of HG on autophagy of HK2 cells. We analyzed the protein expression of LC3, a marker of autophagy by Western blot. The result showed a slight increase in the expression of LC3-II, while the ratio of LC3-II to LC3-I was not significantly affected (Figure 1C). These results were further confirmed by quantitative assessment of autophagic activity with a dual-color DsRed-LC3-GFP reporter [23]. We did not find a significant increase in the number of DsRed-LC3 puncta upon HG incubation (Figure 1F). The above results indicate that HG induces increased apoptosis, but causes no significant change in autophagy of HK2 cells.

### Bim expression is increased in response to high glucose (HG) in HK2 cells

**Figure 2:**
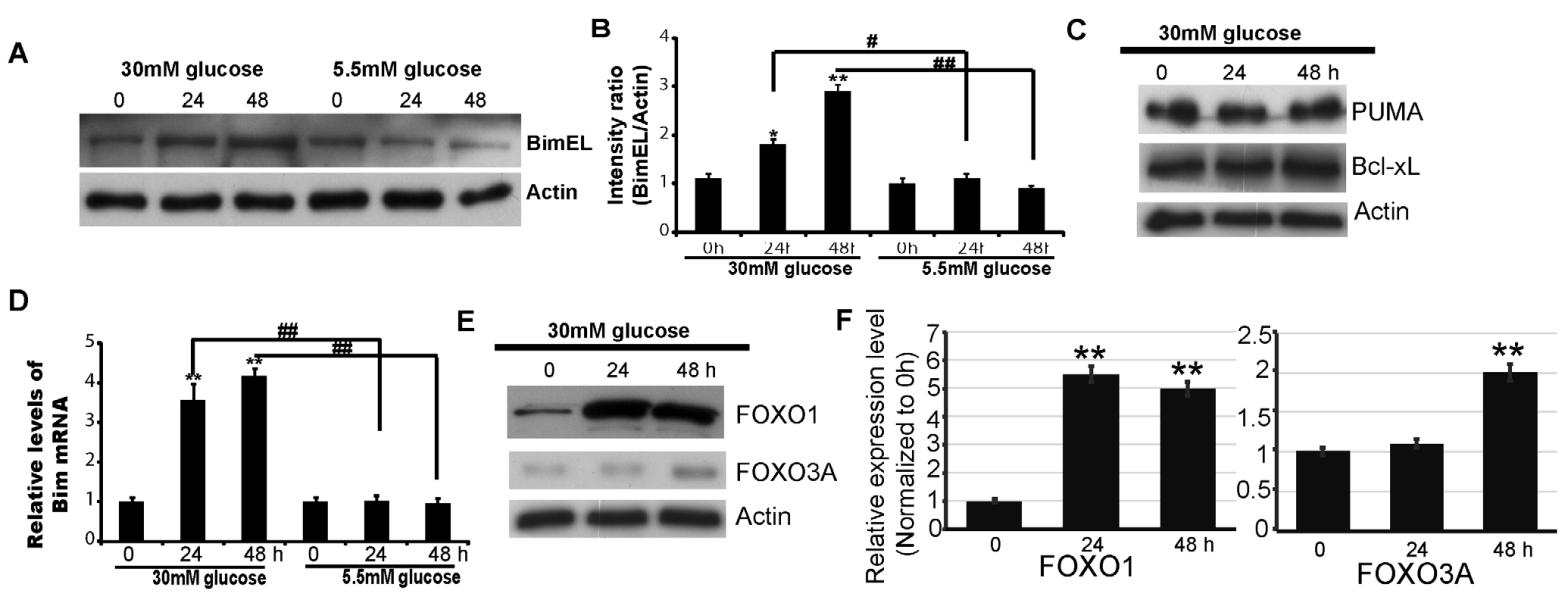
Bim expression is increased by High glucose treatmentin HK2 cells HK2 cells werepretreated with 30mM or 5.5mM glucose for indicated times. **A**. up: Imunobloting analysis of Bim and Actin (The protein are from the same sample of Fig1c). down: BimEL/Actin immunoreactivityintensity is quantitated by densitometric analysis, and optical density values are expressed as a ratiobetween the BimEL and Actin. Data shown are the mean ± s.e.m. *n* = 3. *P < 0.05, **P < 0.01, relative to the 0h group; one-way ANOVA. ^#^*p* < 0.05. **B**. Imunobloting analysis of Bim and Actin in HK2 cells treated with 30mM glucose. **C**. Imunobloting analysis of PUMA, Bcl-xL and Actin in HK2 cells treated with 30mM glucose. **D**. Relative mRNA levels of Bim in HK2cells analyzed byreal-time RT-PCR. Data are shown as the mean ± s.e.m. *n* = 3; *P < 0.05, **P < 0.01,compared with0 h group; Student's t-test. ^##^*p* < 0.01; relative to 5.5mM group; Student's t test. **E**. Imunobloting analysis of FOXO1, FOXO3a and Actin in HK2 cells treated with 30mM glucose. **F**. Quantification of the relative (rel.) levels of FOXO1 and FOXO3A; Data were shown as the mean ± s.e.m. *n* = 3; **P < 0.01, compared with 0 h group; one-way ANOVA.

Bim has three major isoforms: the extra-long form of Bim (BimEL), the long isoforms of Bim (BimL) and the short isoform of Bim (BimS) [24]. These isoforms differ in size and have different apoptotic activity. BimEL is the main isoform and the key effecter molecule in apoptosis regulation. Western blot was performed to determine the involvement of BimEL in HG-induced apoptosis of renal proximal tubule epithelial cells. As shown in Figure 2A, HG treatment increased the expression of the BimEL protein(*P* < 0.05, Figure 2A, 2B), while normal glucose treatment did not. The expressions of other Bcl-2 family members, PUMA and Bcl-xL, were also investigated and no significant changes were found upon HG treatment (Figure 2C).

To determine whether upregulation of Bim expression was the result of enhanced transcription, the mRNA level of Bim was analyzed by Real-time-PCR. A significant increase of Bim mRNA was detected upon HG treatment(*P* < 0.05, Figure 2D). To further investigate the mechanisms on Bim upregulation in HG treatment, we also assessed the expression of FoxO1 or FoxO3A, the transcription factors that can regulate Bim expression [25–30]. Interestingly, both of them were increased upon HG treatment(Figure 2E). The results suggest the involvement of FoxO1 and FoxO3A in Bim upregulation by HG.

### Bim silenced cells are protected from HG-mediated apoptosis

**Figure 3:**
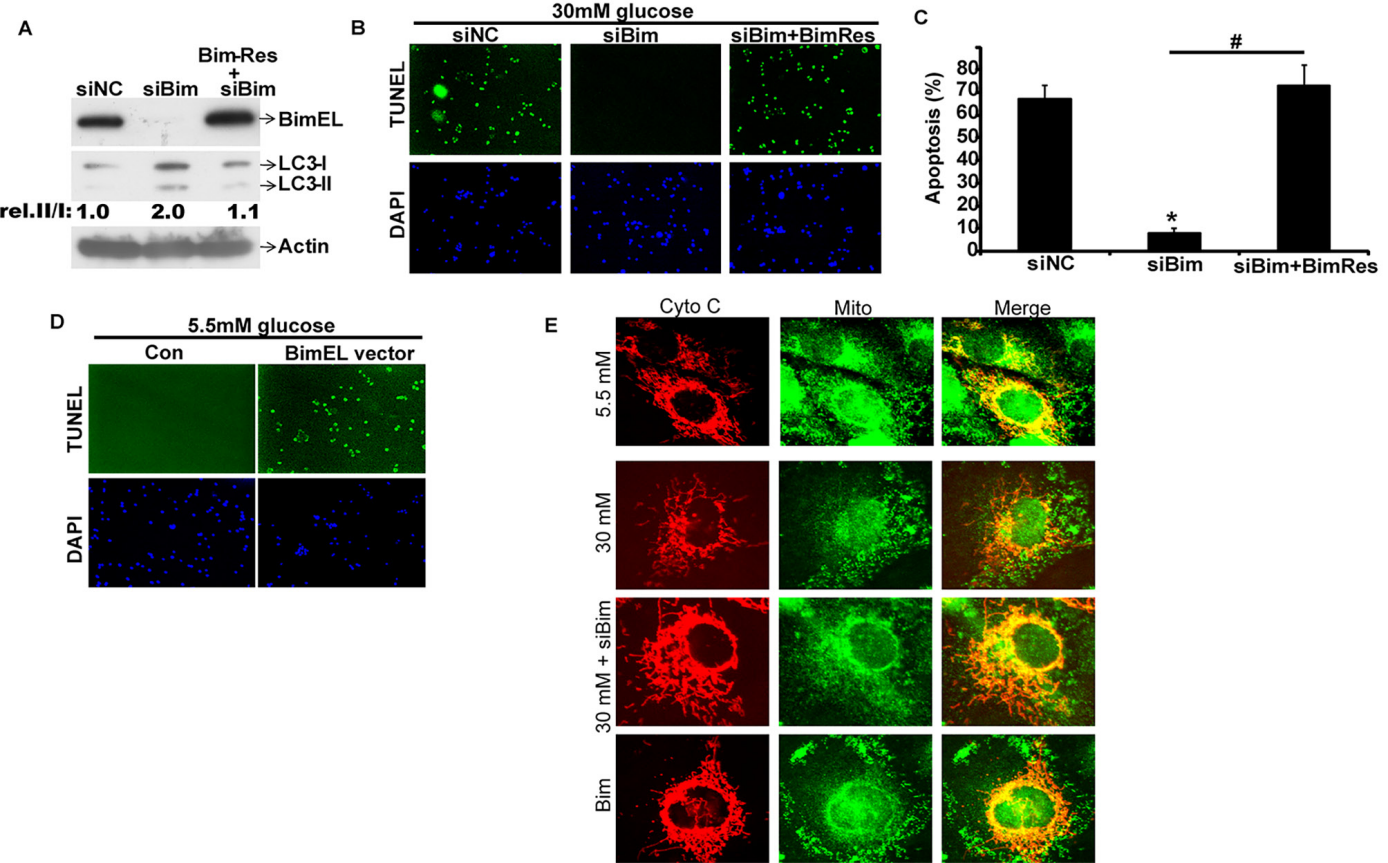
BIMreduced cells by siRNA are protected from HG-mediated apoptosis **A**. HK2 were transfected with siNC, siBim RNA or BimRes plasmidas indicated.Two days later, the cells were lysed.Protein levels of Bim, LC3 and Actin were examined by immunoblotting. Quantification of the relative (rel.) levels of LC3-II/LC3-I was shown with the ratio of 1.0 being assigned to siNC-transfected cells. **B**. HK2 were transfected with siNC, siBimRNA or BimRes plasmidas indicated. Two days later, the cells were treated with 30mM glucose for 48h. Apoptosis was determined by TUNEL staining (green dots) and doubly stained with DAPI (blue dots). **C**. The percent of cell death was quantified by dividing the number of apoptotic nuclei to a population of 1,000 counted cells per condition. Graphs represent means ± s.e.m. *n* = 3; *P < 0.05, relative to the siNC group; #*P* < 0.05, relative to the siBim group; one-way ANOVA. **D**. HK2 were transfected with BimEL plasmidas indicated. Two days later, apoptosis was determined by TUNEL staining (green dots) and doubly stained with DAPI (blue dots). **E.** Cells transiently expressing Ds Red-Mito were treated with indicatied conditions, and the release of Cytochrome C from mitochondria to cytosol was determined based on the overlays of Cytochrome C and Ds Red-Mito fluorescence images.

To investigate the function of Bim in HG-induced apoptosis, we silenced endogenous Bim expression by siRNA transfection. Endogenous Bim expression was reduced by 79% after siRNA transfection (western blot, *n* = 3, *P* < 0.05, Student's t-test, Figure 3A). In Bim-silenced group, HG treatment could not trigger apoptosis of cells anymore, as compared to the significant increase of apoptosis in siNC (Negative control) group (P < 0.05, Figure 3B, 3C).

To get a better understanding on Bim, siRNA-resistant Bim vector was constructed to express BimEL protein (Bim-res), containing silent mutations in the mRNA region targeted by the siRNA. As shown in Figure 3A, endogenous Bim was sensitive to the siRNA, but Bim-res encoded by the cDNA containing mismatches was abundantly expressed in the presence of Bim-siRNA. The response to HG upon apoptosis in the Bim-siRNA transfection group was rescued by the expression of the Bim-res (*P* < 0.05, Figure 3B, 3C). The above results indicated that the upregulated Bim expression was responsible for the apoptosis caused by HG. Furthermore, we found that the overexpression of BimEL was sufficient to cause apoptosis of HK-2 cell even in normal glucose conditions (*P* < 0.05, Figure 3D).

Bim is reported to be the ‘activator’ for Bax activation and mitochondrial apoptosis. Overexpression of Bim leads to the release of cytochrome C and apoptosis [31]. To confirm whether Bim plays the same role in HG-induced apoptosis, we studied the localization of cytochrome C. The colocalization of cytochrome C and mitochondria was observed by both Dsred-Mito plasmid and coupled-Alex 488 cytochrome C antibody. In normal glucose group, cytochrome C colocalized with the mitochondria, while in HG group, cytochrome C did not colocalize with the mitochondria. The result suggests that cytochrome C is released from mitochondria in HK2 cells undergoing apoptosis induced by HG. Importantly, when Bim expression was knocked down, HG could not induce cytochrome C release from mitochondria (Figure 3E).

**Figure 4:**
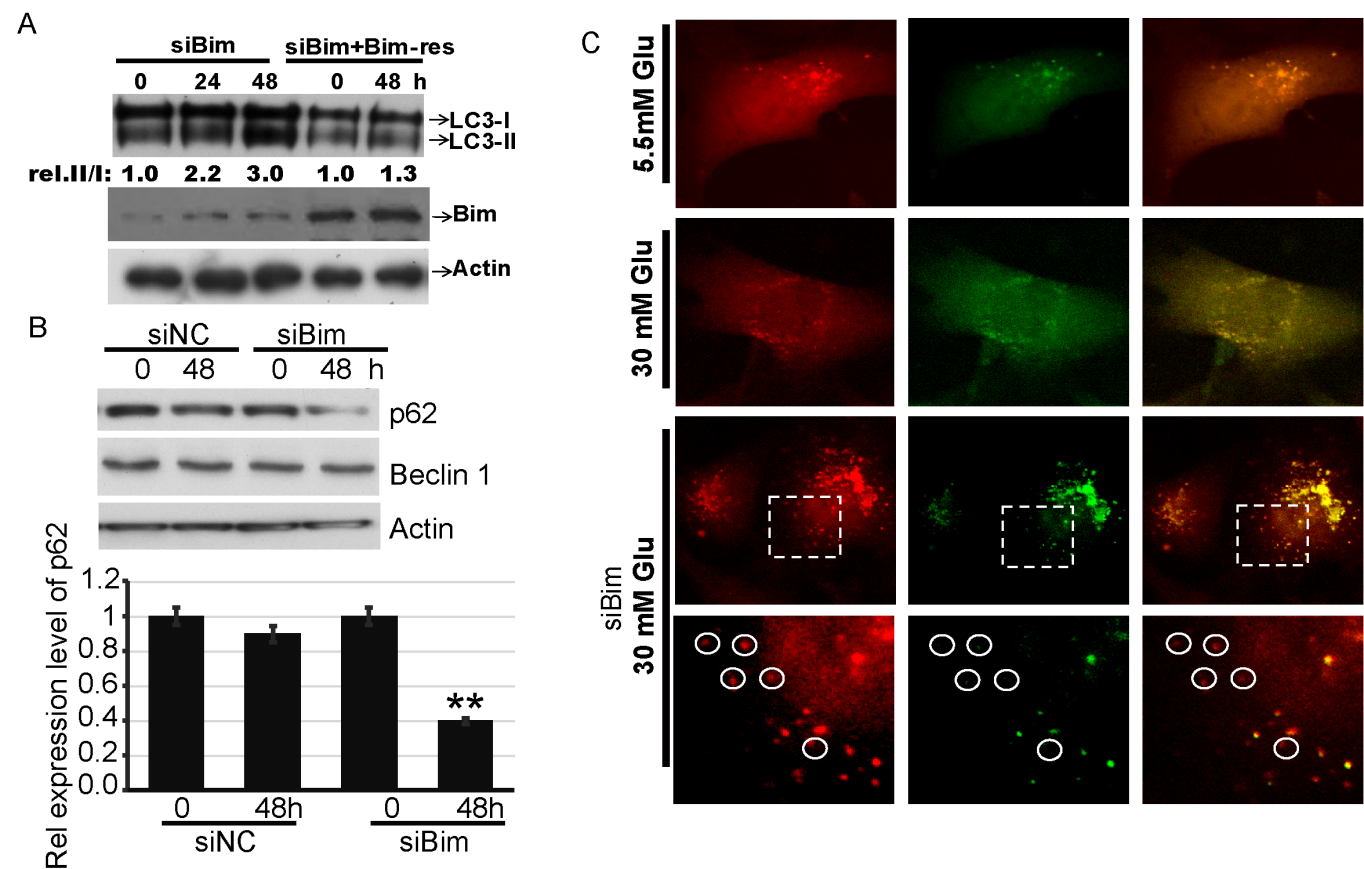
HG promotes autophagy of cells transfected with Bim siRNA **A.** HK2 transfected with indicated plasmid were treated with 30mM glucose for indicated times. Protein levels of LC3 and Actin were examined by immunoblotting. Quantification of the relative (rel.) levels of LC3-II/LC3-I is shown with the ratio of 1.0 being assigned to 0hgroup of siBim-transfected cells. **B**. Up: HK2 were transfected with siNC orsiBim RNA as indicated. Two days later, the cells were lysed. Protein levels of p62, Beclin1 and Actin were examined by immunoblotting. Down: Quantification of the relative (rel.) level of p62; Data are shown as the mean ± s.e.m. *n* = 3; **P < 0.01, compared withsiBim-0 h group; student's t-test. **C**. A549 cells were transfected with Dsred-LC3-GFP for 48 h, followed by treatment with or without high glucose (50 μM). Fluorescence of LC3 was recorded after 48h by fluorescence microscopy.

To investigate whether Bim is involved in autophagy in HK2 cells under HG conditions, the protein expression of LC3 was investigated by western blot. As shown in Figure 4A, the silence of Bim resulted in a remarkable increase of LC3-II and the ration of LC3-II to LC3-I.The increase could be blocked by transfection of siRNA-resistant Bim (*P* < 0.05, Figure 4A). The protein level of p62 (also called sequestosome 1) is normally degraded by lysosomal proteases through interaction with LC3-II during autophagy. As shown in Figure 4B, the level of p62 decreased significantly in Bim knockdown group upon HG treatment. The protein level of Beclin1 was also investigated, and no significant change was found in Bim knockdown group(Figure 4B), which was consistent withprevious report (S. Luo et al, 2012). Cell imaging was further done with Dsred-LC3-GFP reporter construct. The GFP signal was quenched under acidic pH in autophagolysosomes. We used this construct as fluorescent sensor to simultaneously analyze autophagosomes and autophagolysosomes. As shown in Figure 4C, HK 2 cells displayed a steady-state of low autophagic activity when growing in NG or HG media. However, when the cell underwent Bim deprivation, HG significantly activated autophagy. In summary, our results suggested that Bim downregulation induces autophagy, and HG could trigger autophagy but not apoptosis upon Bim-depletion in HK2 cells.

### Autophagy inhibitor 3-MA increases the injury of high glucose in Bim knockdown cells by re-triggering apoptosis

**Figure 5:**
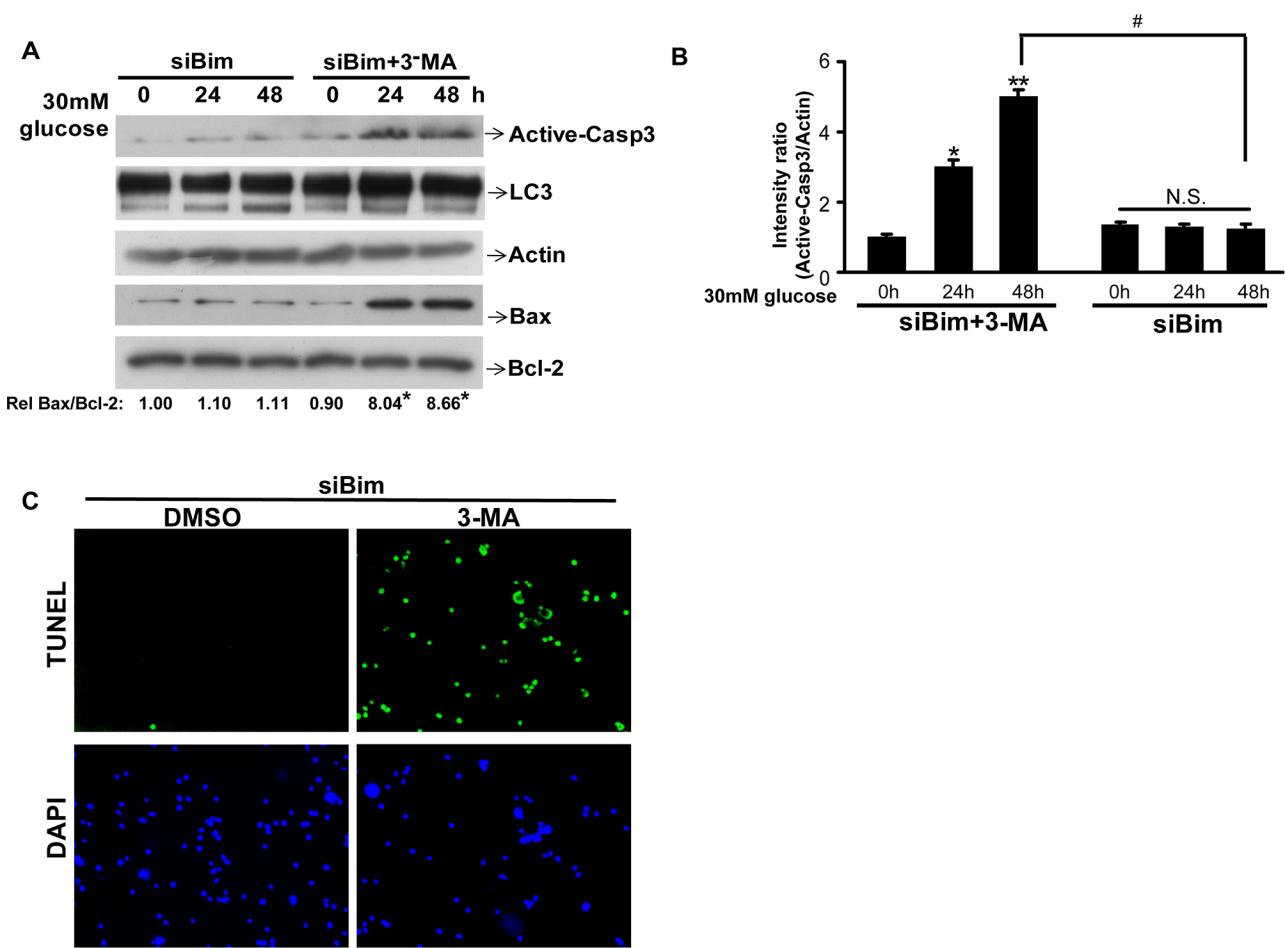
Autophagy inhibitor 3-MA worsens theinjury of high glucose in Bim reduced cells by re-trigger apoptosis **A**. HK2 were transfected with siBim RNA for Two days, then the cells wereco-treated with 3-MA (or DMSO) and 30mM glucose for indicated times and lysed. Protein levels of active-Caspase3, LC3, Actin, BAX and BCL-2 were examined by immunoblotting. Quantification of the expressionratio of BAX/BCL-2 is shown with the ratio of 1.0 being assigned to siBim-0h cells. *P < 0.05; one-way ANOVA. **B**. Active-Caspase3/Actin immunoreactivityintensity was quantitated by densitometric analysis, and optical density values were expressed as a ratiobetween the Active-Caspase3 and Actin. Data shown are the mean ± s.e.m. *n* = 3. *P < 0.05, **P < 0.01, ^#^*P* < 0.05, Student's t test.N.S. indicates not significant. **C**. HK2 were transfected with siBim RNA for Two days, then the cells wereco-treated with 3-MA (or DMSO) and 30mM glucose for 48h. Apoptosis was determined by TUNEL staining (green dots) and doubly stained with DAPI (blue dots).

To examine the possible link between autophagy and apoptosis in HK2 cells responding to HG, Bim knockdown cells were treated with 3-methyladenine (3-MA), an inhibitor of autophagy via inhibiting autophagosomes formation [32]. The expression of apoptosis marker, Active-Capase 3, and the autophagy marker LC3-II were evaluated. To our surprise, in the siBim transfection group, HG induced autophagy significantly, while 3-MA retriggered apoptosis in Bim knockdown cells (*P* < 0.05, Figure 5A, 5B). We also performed TUNEL assay. The result indicated that when Bim was knocked down, 3-MA itself did not trigger apoptosis in NG condition; however, in HG condition, although autophagy was blocked by 3-MA, the cells still showed a higher TUNEL-positive rate (Figure 5C). The results suggest that a Bim-independent apoptosis pathway might be active in response to HG conditions.

### HG induces Bim dependent apoptosis not only in HK2 cells, but also in primary proximal tubule epithelial cells

**Figure 6:**
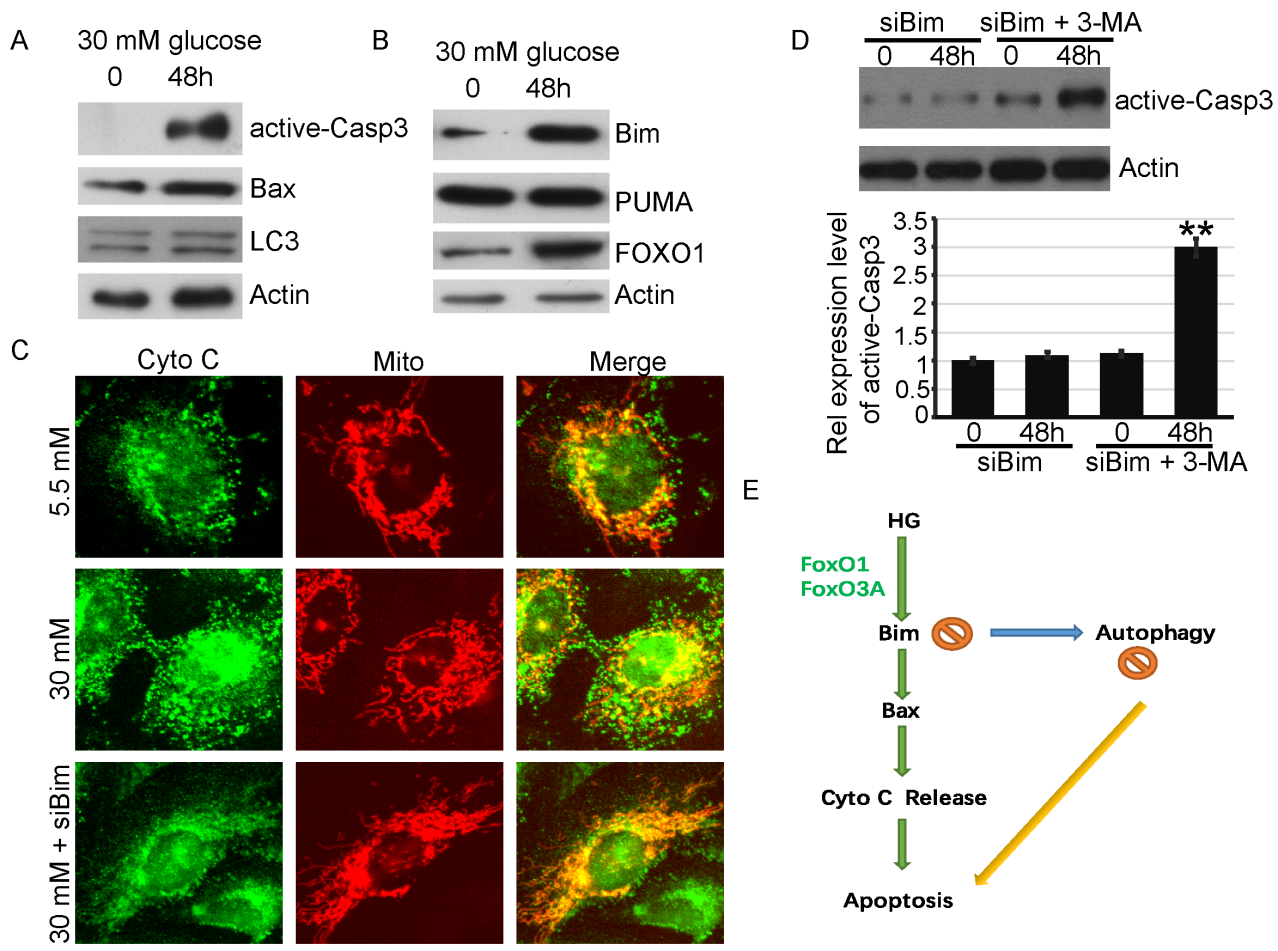
HG also induces Bim dependent apoptosis in primary proximal tubule cells **A.** Primary proximal tubule cells were treated with 30mM glucose for indicated times. Protein levels of active-Caspase3, LC3, BAXand Actin, were examined by immunoblotting. **B.** Primary proximal tubule cells were treated with 30mM glucose for indicated times. Protein levels of Bim, LC3, PUMA, FOXO1and Actin, were examined by immunoblotting. **C**. Cells transiently expressingDsRed-Mito were treated with indicatied conditions, and the release of Cytochrome cfrom mitochondria to cytosol was determined based on the overlays of Cytochrome cand DsRed-Mito fluorescence images. **D**. Primary proximal tubule cells were transfected with siBim RNA for Two days, then the cells were co-treated with 3-MA (or DMSO) and 30mM glucose for 48h and lysed. Protein levels of active-Caspase3 and Actin were examined by immunoblotting. Active-Caspase3/Actin immunoreactivityintensity was quantitated by densitometric analysis. Data shown are the mean ± s.e.m. *n* = 3. **P < 0.01, Student's t test. **E**. simplifiedmodeldepicting the working model of HG induce apoptosis in proximal tubule cells.

To further validate our findings, experiments were also done in human primary proximal tubuleepithelial cells. As shown in Figure 6A, HG treatment could also cause apoptosis in human primary proximal tubuleepithelial cells. The upregulation of Bim and its transcription factor FoxO1, were detected (Figure 6B), and our results showed that the expression of both Bimand FOXO1 is increased. HG is associated with the release of cytochrome c from mitochondria, and Bim knockdown blocked it (Figure 6C). When Bim was silenced, HG triggered autophagy but not apoptosis. When autophagy was blocked by 3-MA, there was also Bim-independent apoptosis in primary proximal tubule epithelial cells (Figure 6D). In short, our findings in HK2 cells were reproducible, in primary proximal tubuleepithelial cells.

## DISCUSSION

The prevalence of diabetic nephropathy has been increasing worldwide. Diabetic nephropathy (DN) is the most common cause of end stage renal disease (ESRD). While previous studies indicate that DN is mainly a disease of glomerulapathy [1, 33, 34], a growing body of evidences indicates that the renal proximal tubule epithelial cell (PTEC) plays an important role in the pathogenesis of diabetic nephropathy [35, 36]. Recent studies suggest a role of cell death in the progression of human DN [37, 38]. Autophagic activity in the pathogenesis of DN is associated with an increase in p62/SQSTM1 in both proximal and distal tubule cells of both type 1 and type 2 diabetic animals [19, 39, 40]. While targeting the autophagic pathway and restoring autophagic activity may be renoprotective [18, 19], mechanisms of autophagy and apoptosis involved in diabetic nephropathy remain largely unknown.

It has been reported that Bim works as a molecular link between autophagy and apoptosis, and Bim could reduce autophagy activity [41]. In response to death stimuli, Bim dissociates from the dynein light chain 1 (DYNLL1/LC8), and then initiates BAX/BAK-mediated mitochondria-dependent apoptosis [29, 42]. Bim also inhibits autophagy by interacting with Beclin 1, an autophagy regulator. Recently, it was reported that Bim depletion increases autophagosome synthesis in cells [41].

Our study provides evidence that HG regulates Bim expression through upregulating the transcript factors FOXO3A and FOXO1. The overexpression of Bim further triggers apoptosis in HK2 cells. The HG-induced Bim upregulation causes the release of cytochrome C from mitochondria, which may be the reason for apoptosis. This process can be blocked by reducing Bim expression. Bim deprivation switch cells into autophagy upon HG treatment. Furthermore, autophagy inhibitor 3-MA in Bim silenced cells could retrigger apoptosis in a Bim-independent manner, such as through BAK or PUMA [43, 44]. Further study is still needed to address this question. Our study indicated that Bim played a fate-destination role and was a sufficient regulator to switch autophagy or apoptosis in HK2 cells. Those results provide new insights into the mechanism of HG-induced injury in HK2 cells.

First, we showed that HGinduced apoptosis via upregulating the expression of Bim protein,but not bring a significant change in the baseline level of autophagy in HK2 cells. The increase of Bim expression was caused by the ugregulation of transcription factors, FOXO1 and FOXO3A. Bim expression initiates BAX/BAK-mediated mitochondria-dependent apoptosis [31]. Our result was not consistent with Gou's and Lee's studies [45, 46], which reported that high glucose increased autophagy. The difference might be caused by different methodology of the experiment.

Second, we showed that the silence of Bim promotes autophagy of HK2 cells and protects them from HG-mediated apoptosis. When Bim was overexpressed, the cells showed increased apoptosis, even under normal glucose conditions. The results indicate that Bim is involved in switching HK2 cells between apoptosis and autophagy. While preventing apoptosis in some cells by silencing Bim does not necessarily mean that those cells will undergo autophagy. Activation of another pathway by HG may be also required in order to get induction of autophagy.

Third, we found there is still a Bim-independent apoptosis response for HG when autophagy is blocked. The autophagy inhibitor 3-MA could retrigger apoptosis in Bim knockdown cells. This suggests a Bim-independent apoptosis pathway [44], which is also independent of autophagy.

Given that the impairment of autophagy is implicated in the pathogenesis of DN, deregulation of Bim might be important in DN therapy. It is confirmed that, when Bim is inhibited, HK2 cells could restore autophagy activity and protect themselves from injury induced by high glucose. Therefore, therapeutic strategies might be targeted to reduce the endogenous Bim or inhibit Bim function.

To the best of our knowledge, our study is the first to provide evidence that high glucose induces apoptosis via upregulating Bim expression in proximal tubule epithelial cells, and the upregulation of Bim impairs autophagy activity. Downregulation of Bim restores the autophagy activity of the cells, and protects them from apoptosis. These findings provide new insights into our understanding about how apoptosis and autophagy, the two critical process in DN, relate and interact, which also suggests a new target for the prevention or treatment of diabetic nephropathy.

## MATERIALS AND METHODS

### Cell culture

HK-2 cells, a proximal tubule epithelial cell line (American Type Cell Collection, Rockville, MD), were cultured in the RPMI 1640 medium containing 10% FBS, 11.1 mM glucose, 100 units/ml penicillin-streptomycin (Sigma, St. Louis, MO) at 37 °C, 5% CO2, and 95% humidity.

### Culture of human primary proximal tubuleepithelial cells

Human primary proximal tubule epithelial cells were purchased from Pricells Company (Wuhan, China) and cultured in a primary proximal tubule epithelial cell culture medium (Pricells, Wuhan, China) supplemented with 10% fetal calf serum (Gibco, USA), 100 U/ml penicillin and 100 μg/ml streptomycin.

### Antibodies and reagents

Antibodies in the study were from the following sources: anti-LC3 (Mono) from Cell Signaling Technology (Beverly, MA), anti-Bim (Mono), anti- Cytochrome c, anti-p62, anti-Beclin and anti-caspase3 (ployclonal) from Abcam (Cambridge, MA), anti-β-actin from Sigma (St. Louis, MO); anti-Bax, anti-FOXO1, anti-FOXO3A and anti-BCL-2 (ployclonal) from Proteintech Group (Chicago, IL). All secondary antibodies (ployclonal) were from Jackson ImmunoResearch Laboratories Inc (West Grove, PA). Lipofectamine 2000 transfection reagents were from Invitrogen (Carlsbad, CA). Unless indicated, other reagents were from Sigma (St. Louis, MO).

### Quantitative RT-PCR

The mRNA expression of Bim induced by HG was measured by quantitative RT-PCR. HK2 cells were stimulated with 30mM glucose for the indicated times. Afterward, cells were washed with PBS and followed by RNA extraction. Total RNA was extracted with TRNzol-A+ RNA isolation reagent (TIANGEN), according to the manufacturer's instructions. Reverse transcription was performed with 1 μg of total RNA and RevertAid First Strand cDNA Synthesis Kit (Fermentas). Primer sequences were used as follows: Bim forward primer: 5′- ATT ACC AAG CAG CCG AAG AC -3′ and reverse primer: 5′- TCC GCA AAG AAC CTG TCA AT -3′; β-actin forward:5′- TGA CGT GGA CAT CCG CAA AG -3′ and reverse: 5′- CTG GAA GGT GGA CAG CGA GGT-3′. Quantitative RT-PCR was performed in a cycler (MyiQ2, Bio-Rad) using SYBR green (Roche). The Bim mRNA levels were normalized by the β-actin expression. Each experiment was repeated three times.

### Plasmid constructs and siRNA

Knockdown of Bim expression was performed by RNA interference using specific siRNA oligonucleotides (Qiagen). The target sequences for Bim were as follows: CGGAGACGAGTTTAACGCTTA. To generate the plasmid encoding Bim siRNA-resistant wild type of Bim-EL, pCAGIG-siRNAR-BimEL (Bim-Res) mutants were made by site-directed mutagenesis (without amino acid change) corresponding to the sequence of Bim siRNA. The PCR product was subcloned into pCAGIG expression vector (addgene) using the XbaI and BamHI sites. The construct was confirmed by DNA sequencing to exclude protein connected to EGFP.

### Immunofluorescence assay

HK2 cells were plated on PDL-coated glass coverslips in six-well tissue culture plates. After 24 hours, the cells were transfected with Dsred-Mito plasmid. After 48 hours, the slides were rinsed with PBS and fixed for 10 minutes with 4% paraformaldehyde in PBS at room temperature. This was followed by permeabilization with 0.05% Triton X-100 in PBS for 15 minutes. After rinsing twice with PBS, the slides were incubated with Cytochrome c antibodies for 1 hour at room temperature, rinsed four times with PBS and incubated with Alex 488 labeled secondary antibodies (1:1000 in 5% normal goat serum) for 1 hour at room temperature. After rinsing twice with PBS, slides were finally mounted on glass cover slips using VECTASHIELD (Vector Laboratories), and images were collected and analyzed on a Nikon confocal microscopy system.

### Assessment of apoptosis

In situ detection of apoptosis was performed on sides by the terminal deoxynucleotidyl transferase (TdT)-mediated dUDP nick-end labeling (TUNEL) technique using an In Situ Cell Death Detection Kit (Roche). HK2 cells were seeded onto sterile glass cover slips in a six-well plate then stimulated with 30mM glucose for the indicated times. The cells were assessed by TUNEL assay, according to the manufacturer's recommendation.

### Western blot

Protein level was measured by western blotting. Briefly, equal amounts of protein were separated by SDS-PAGE electrophoresis and transferred to PVDF membrane (Bio-Rad Inc.). Then the membranes were incubated in a blocking buffer (0.2 mM Tris, 137 mM NaCl, 5% no-fat milk, and 0.1% Tween-20) for one hour and probed at 4 °C overnight with specific primary antibodies. The membranes were rinsed with TBST buffer (0.1% Tween 20, 0.2 mM Tris, and 137 mM NaCl) and incubated with HRP-conjugated secondary antibody (1:5000) for one hour at room temperature, followed by chemiluminescent detection.

### Statistical analysis

All statistical analyses were performed using SPSS (Statistical Product and Service Solutions)19.0 software (from IBM). A Student's t-test was used to assess significance for data within two groups. Multiple statistic comparisons were analyzed using one-way ANOVA and followed by post hoc tests. Data are presented as mean ± SEM, and significance was set at *p* < 0.05.
